# Supplementary material for: Structures of tmRNA and SmpB as they transit through the ribosome
Source: Nat Commun. 2021 Aug 13;12:4909. doi: 10.1038/s41467-021-24881-4 (PMC8363625; doi:10.1038/s41467-021-24881-4)
Supplement: Supplementary file 1 — Supplementary Information [file 41467_2021_24881_MOESM1_ESM.pdf]

Structures of tmRNA and SmpB as they transit through the ribosome

Charlotte Guyomar*[1], Gaetano d'Urso*[1], Sophie Chat[1], Emmanuel Giudice[1], and Reynald Gillet[1]

[1]Univ. Rennes, CNRS, Institut de Génétique et Développement de Rennes (IGDR) UMR 6290, Rennes, France.

Correspondence and requests for materials should be addressed to R.G. (email: reynald.gillet@univ-rennes1.fr) or E.G. (email: emmanuel.giudice@univ-rennes1.fr)

*These authors contributed equally to this work.

Supplementary Figures

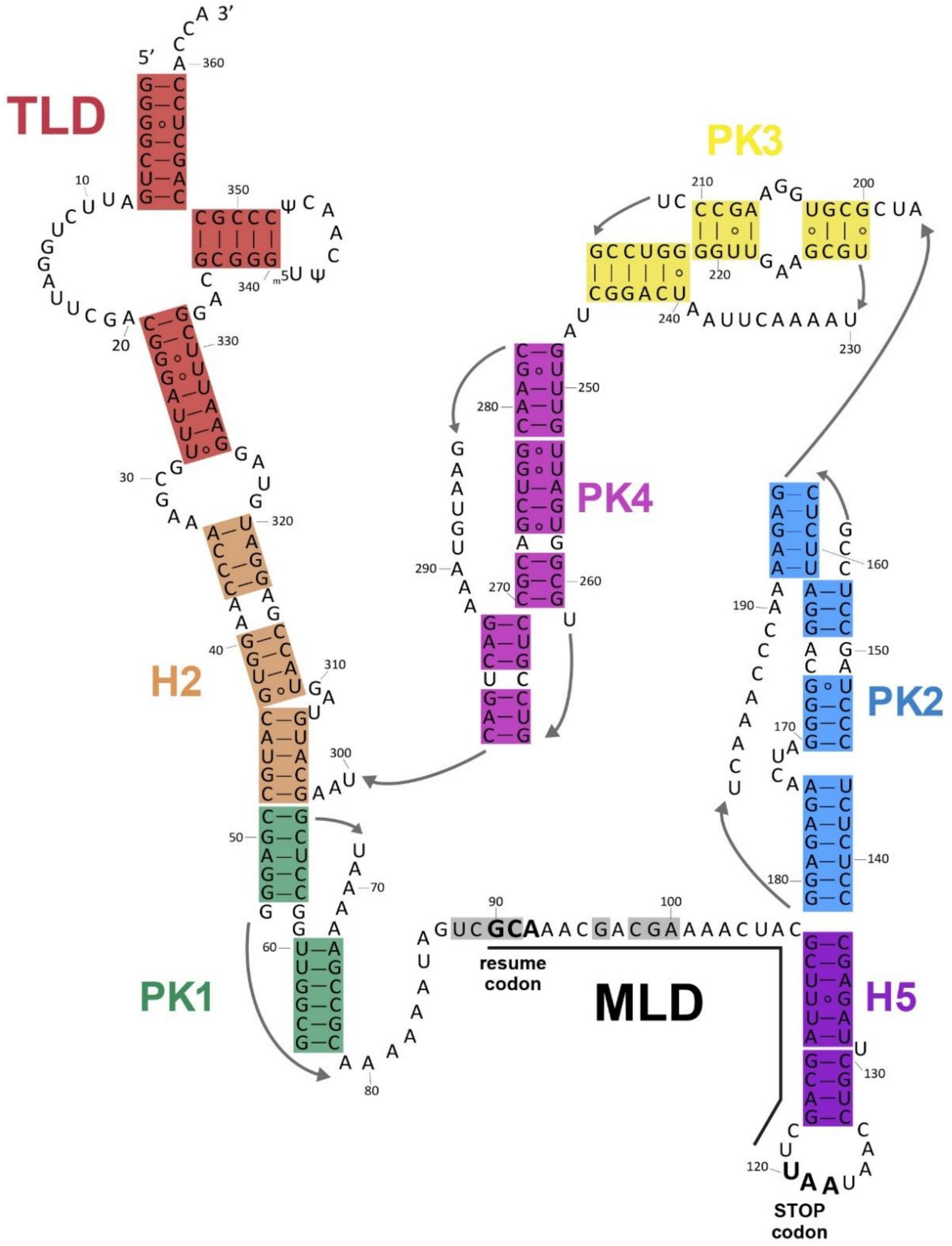

Supplementary Figure 1. Secondary structure of *Escherichia coli* tmRNA. To highlight the different structural domains, the helices in the tRNA-like domain (TLD) are red, the H2 helix is orange, the H5 stem-loop is purple, and the four pseudoknots (PK1 to PK4) are green, light blue, yellow, and pink, respectively. The mRNA-like domain (MLD) is underlined in black, with the putative transient central hairpin highlighted in grey. The resume codon (positions 90 to 92) and the stop codons (positions 120 to 122) are indicated. The grey arrows indicate the connection between consecutive nucleotides, and nucleotides are numbered in increments of ten.

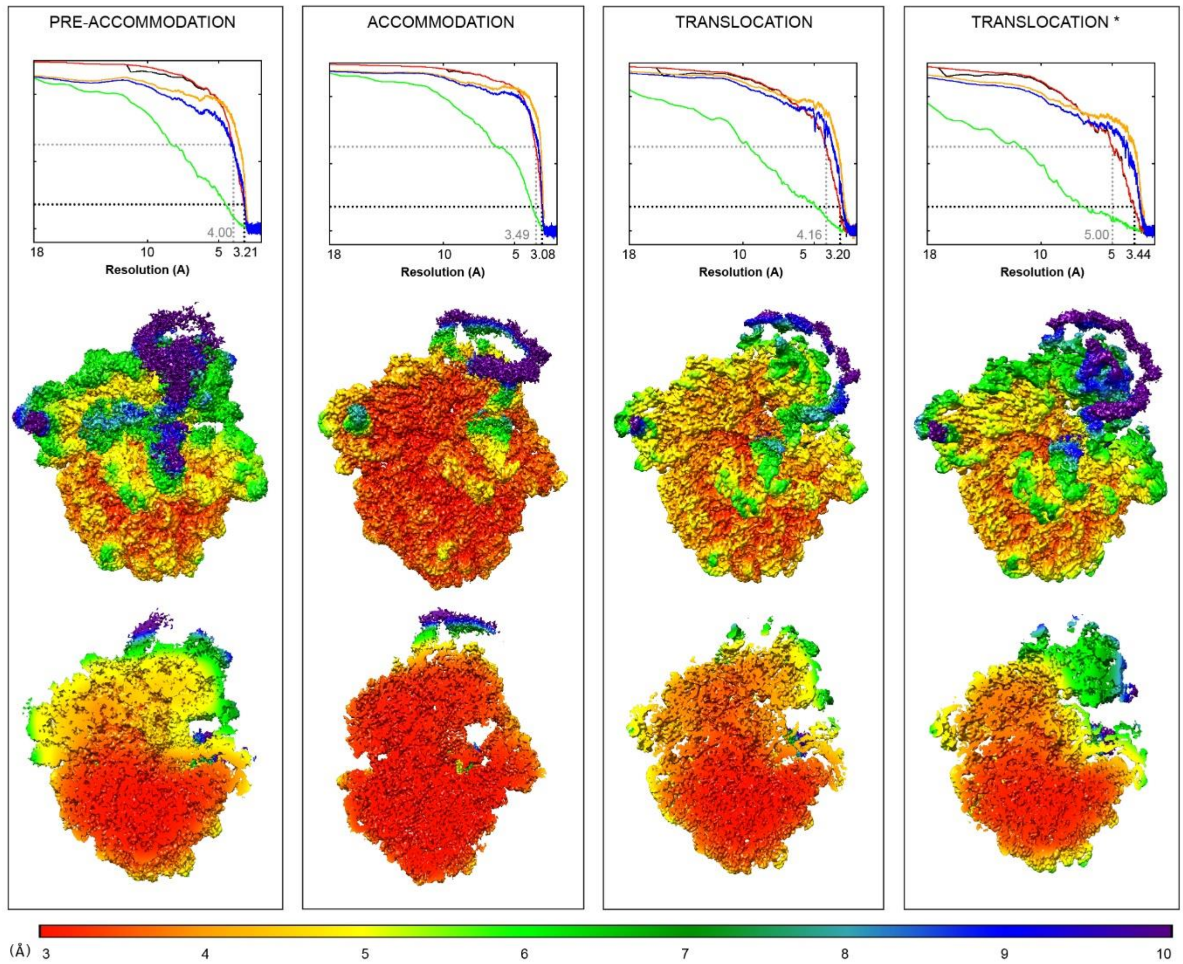

Supplementary Figure 2. Evaluating map quality for the consensus density maps. *Top*, Fourier shell correlation (FSC) curves calculated between the unmasked (green), masked (red), or solvent-corrected (black) half-maps, and between the atomic model and the unmasked (orange) or masked (blue) consensus-sharpened maps. Dashed lines indicate $FSC = 0.143$ (black) and 0.5 (grey). Corresponding resolutions calculated for the solvent-corrected half-maps are indicated. *Middle*, Front view of the consensus-sharpened density maps, which are coloured according to the local resolutions as computed with ResMap[1]. *Bottom*, Same as middle view, but sliced halfway through the maps.

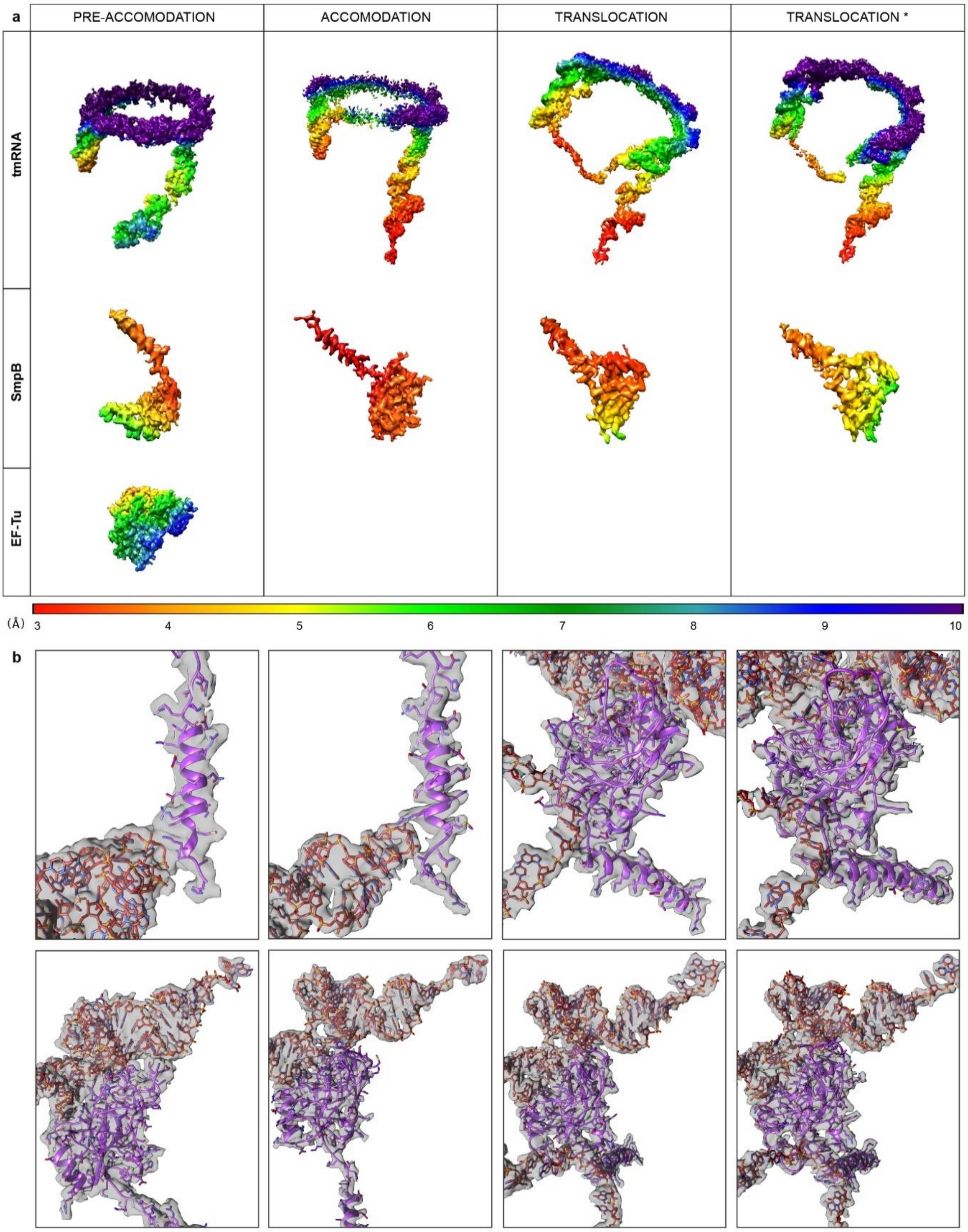

Supplementary Figure 3. Local resolutions of each of the individual components of the *trans*-translation complex. (a) Consensus-sharpened maps of tmRNA, SmpB, and EF-Tu are coloured according to the local resolutions as computed with ResMap[1]. **(b)** A representative sample of the maps showing the densities along with the models of SmpB, the tip of H5, the TLD, and the MLD.

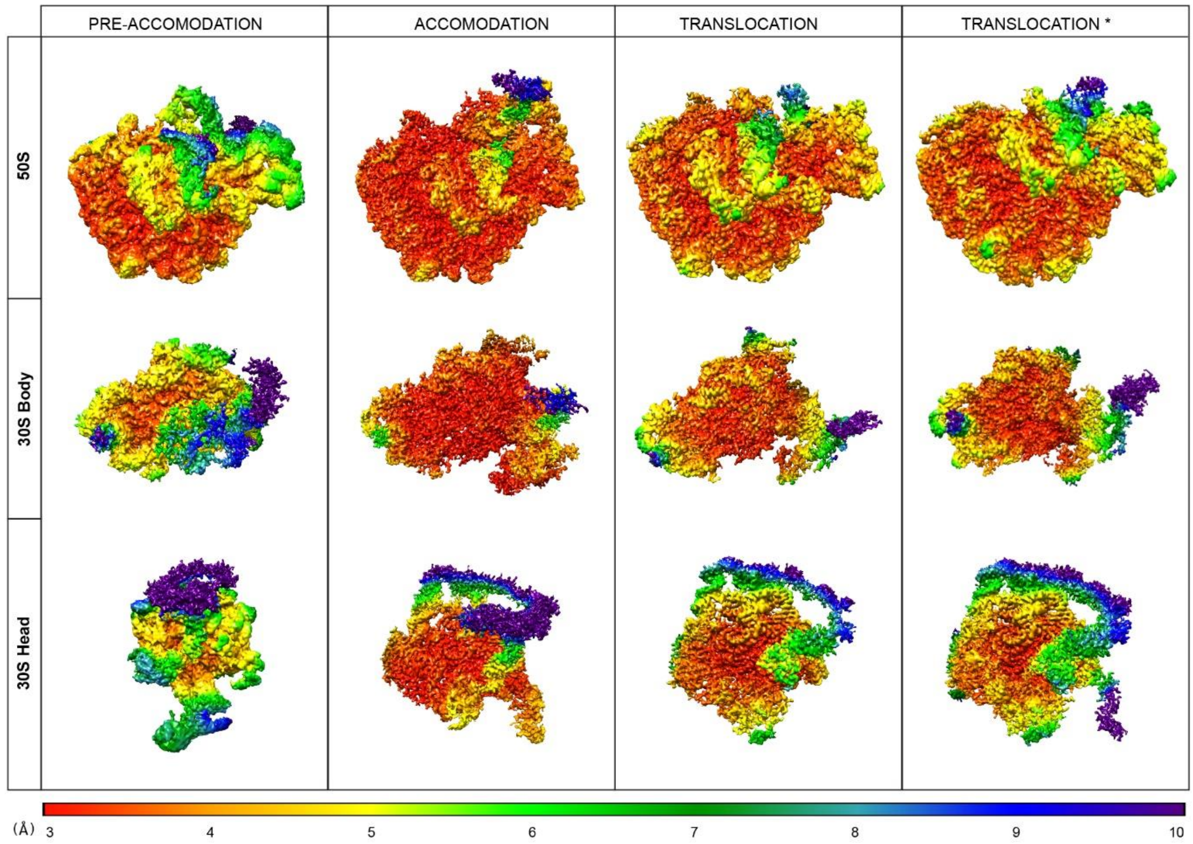

Supplementary Figure 4. Evaluating map quality for multi-body refinement. Sharpened-density maps of the large ribosomal subunit, small ribosomal subunit body, and small ribosomal subunit head regions are coloured here according to the local resolutions as computed with ResMap[1].

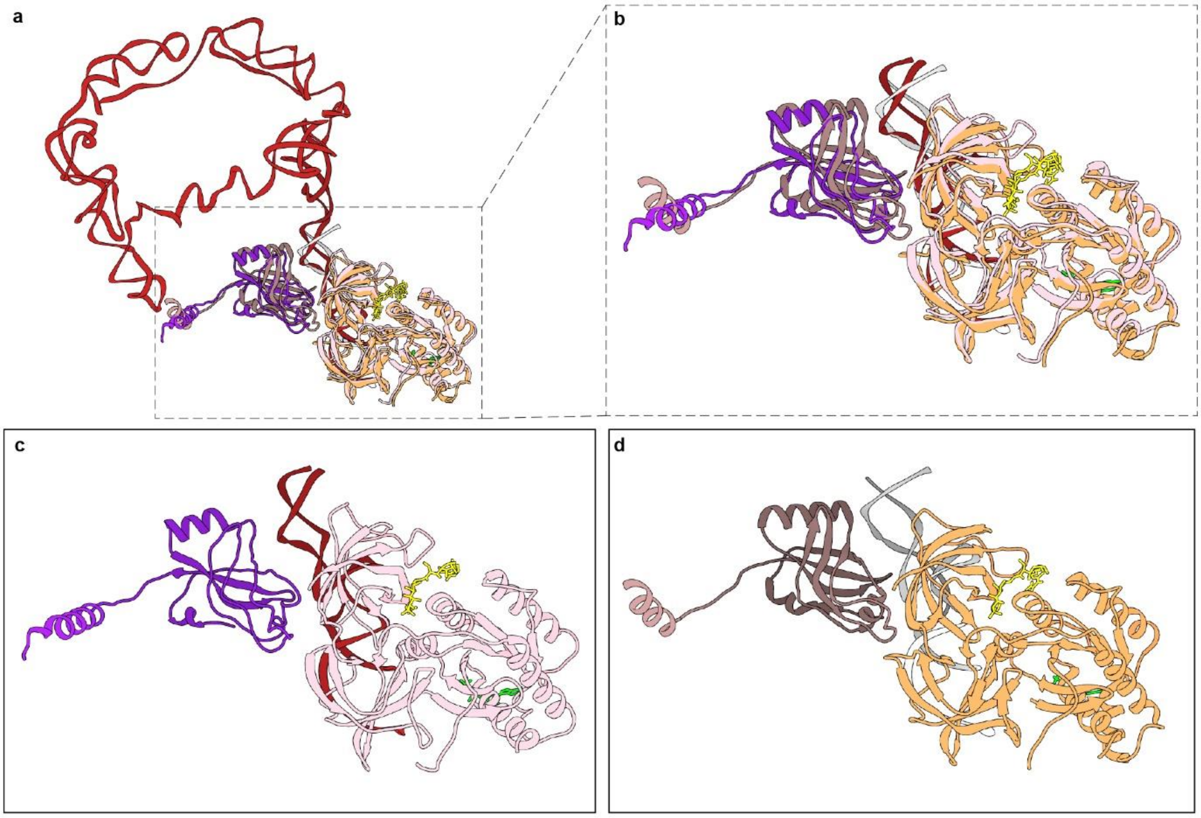

Supplementary Figure 5. Structural comparison between the Ala-tmRNA-SmpB-EF-Tu·GDP quaternary complex observed in the pre-accommodation state and a crystal structure of Ala-tmRNAΔ^{m}·SmpB·EF-Tu·GDP. (a) Global view of the overlay of our structure (tmRNA is red, SmpB is purple, and EF-Tu is light pink) and the structure published by Neubauer *et al.*[2] (PDB code 4V8Q, with tmRNA grey, SmpB dark pink, and EF-Tu orange) obtained after rigid-body fitting their atomic model (including the ribosome) into our electron density map. The RMSD between the two complexes calculated for the P and Cα is 3.23 Å, with SmpB (Cα-RMDS^{SmpB} = 4.1 Å) accounting for the biggest difference between the two structures. In both, GDP is green and kirromycin is yellow. **(b)** Close-up of the shared areas of the two structures, which are then shown separately in **(c)** and **(d)**.

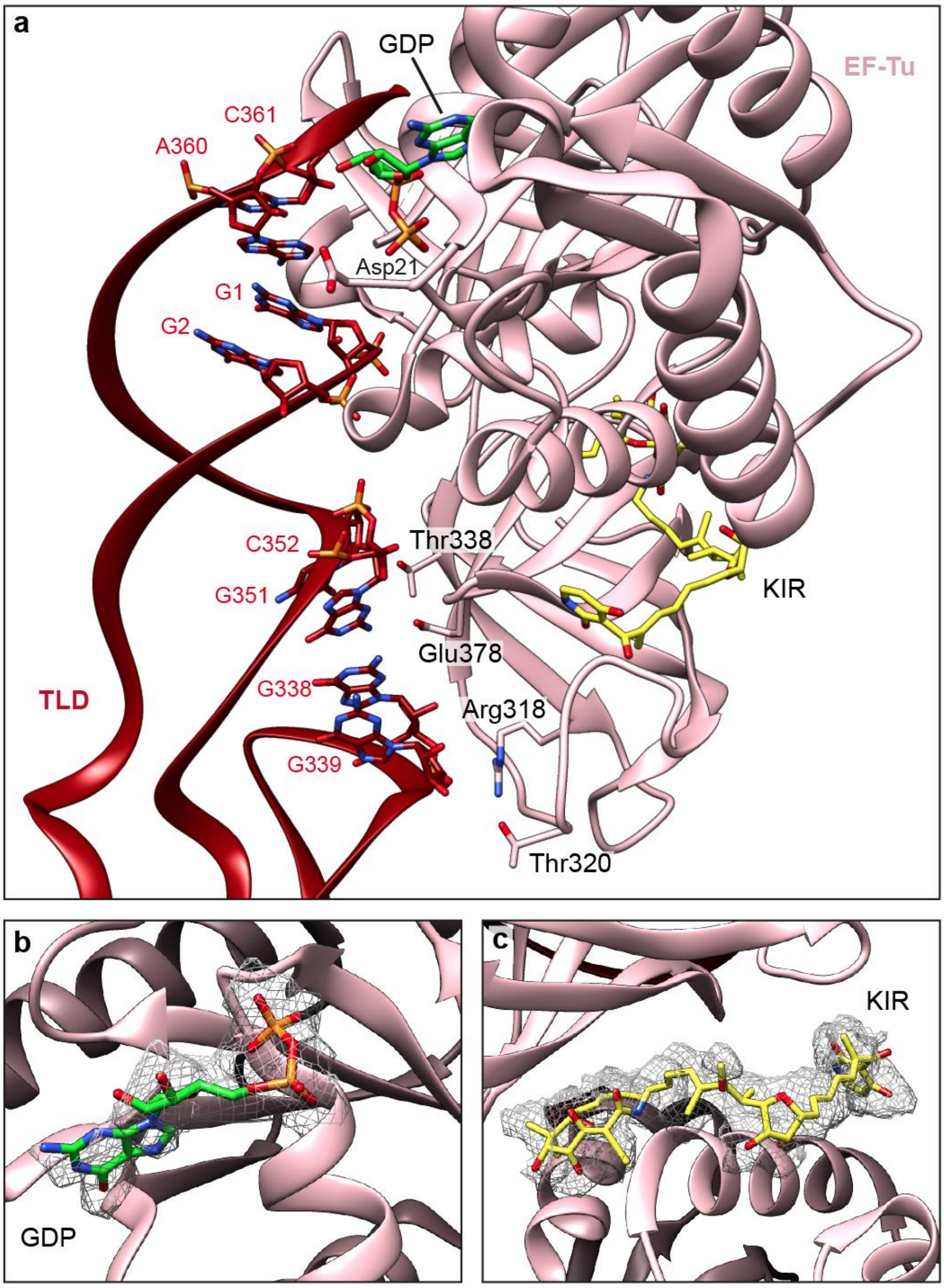

Supplementary Figure 6. Interactions between EF-Tu and the tRNA-like domain (TLD) of tmRNA**. (a)** Details of the contacts between the TLD (red) and EF-Tu (pink). All residues and nucleotides within 4 Å of each other are indicated. **(b-c)** Focus on the densities of **(b)** GDP (green) and **(c)** kirromycin (KIR, yellow). The cryo-electron density maps of the GDP and KIR are shown as grey meshes.

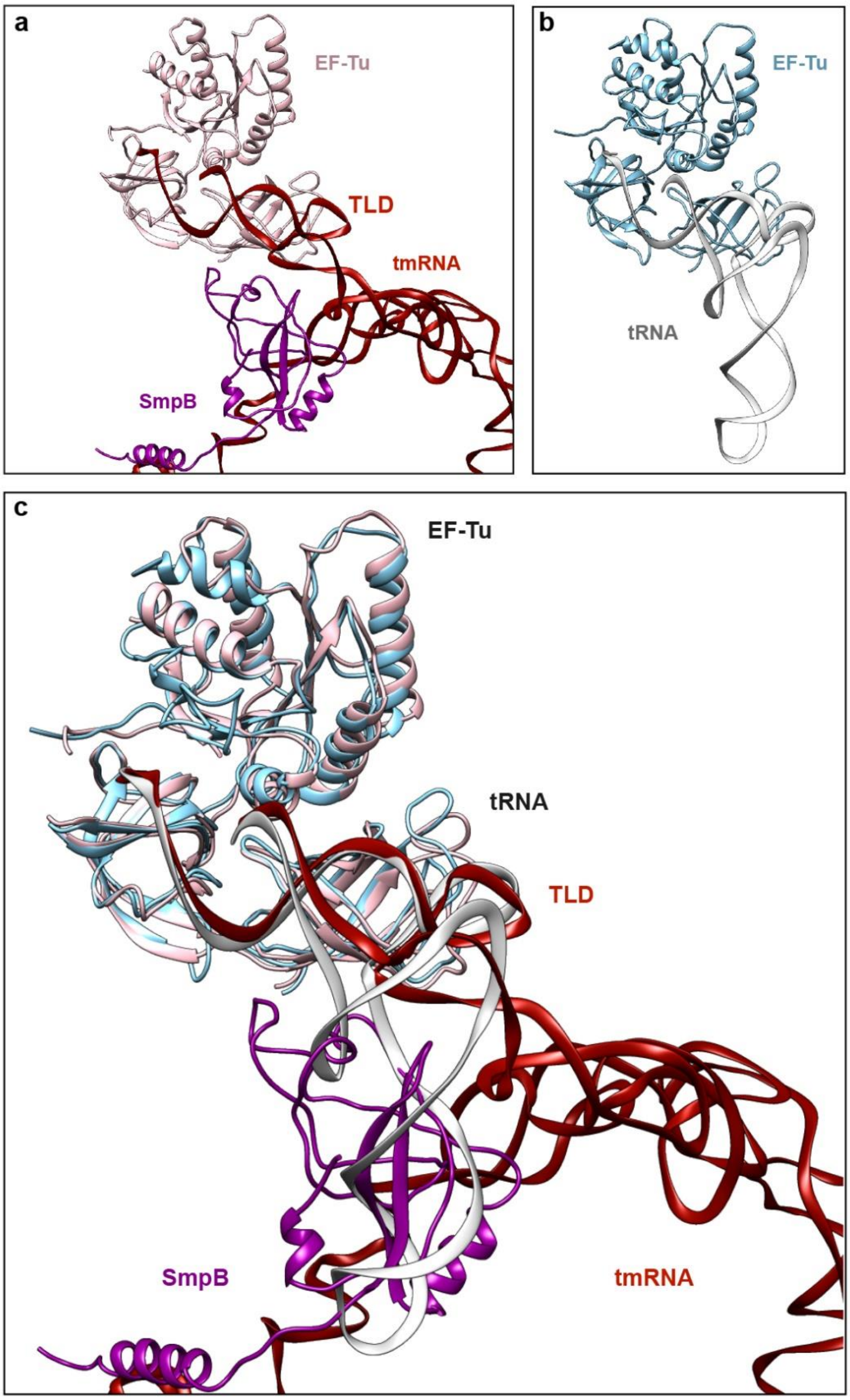

Supplementary Figure 7. Structural comparison between the tmRNA-SmpB-EF-Tu·GDP quaternary complex and a canonical tRNA-EF-Tu·GDP ternary complex. **(a)** Overview of the tmRNA-SmpB-EF-Tu·GDP quaternary complex. The tmRNA is red, SmpB is purple, and EF-Tu is pink. **(b)** Overview of the canonical tRNA-EF-Tu·GDP ternary complex[3] (PDB code 4V5L). EF-Tu is light blue and tRNA is white. **(c)** Overlaying the two structures highlights the similarities between the TLD and the upper part of the tRNA, while also showing that SmpB replaces the tRNA anticodon stem-loop.

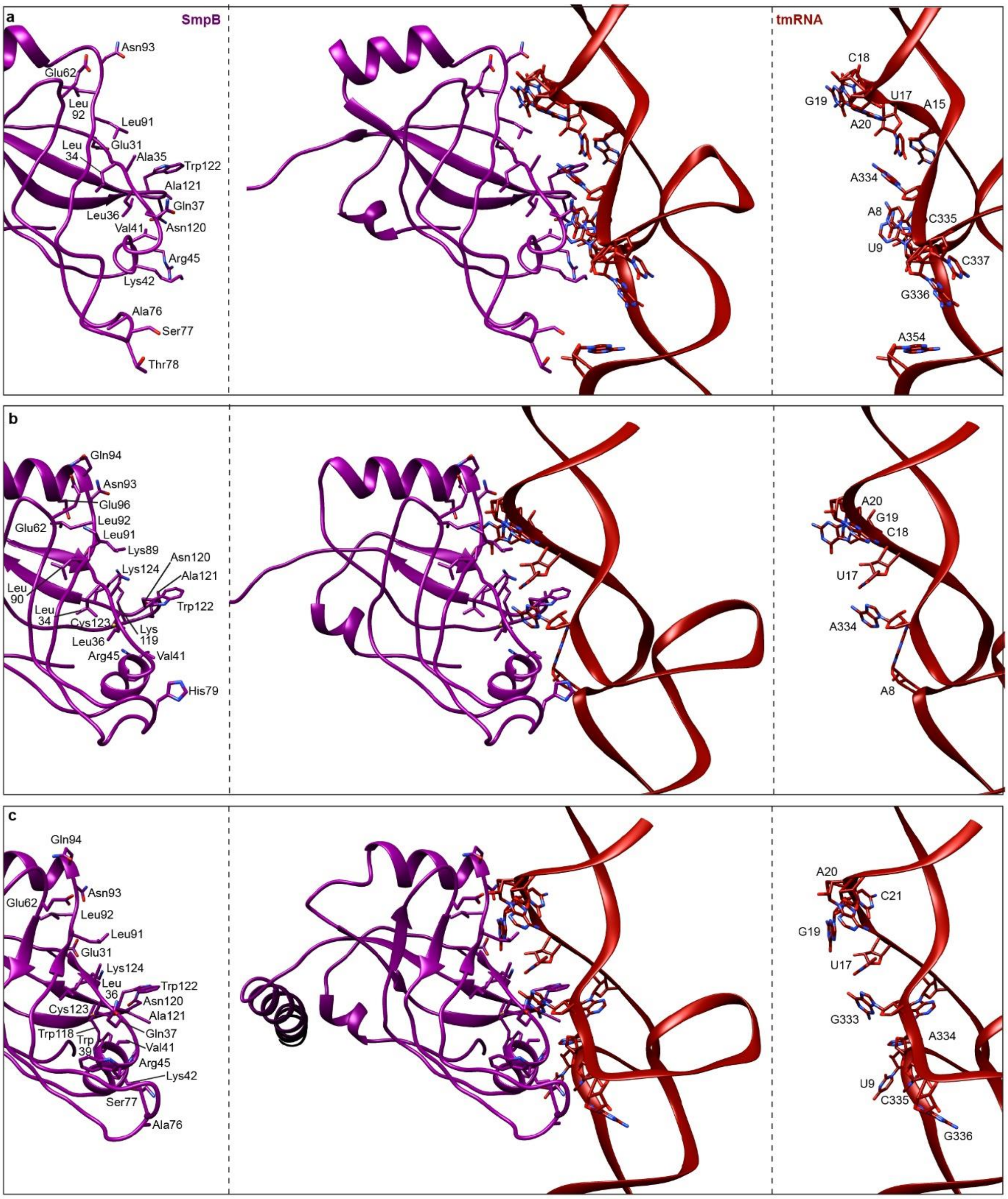

Supplementary Figure 8. Details of the interactions between the SmpB body and the tmRNA TLD during *trans*-translation. Shown are the **(a)** pre-accommodation, **(b)** accommodation, and **(c)** translocation states. *Left*, Focus on the SmpB residues that are within 4 Å of the TLD. *Middle*, Overview of the interaction between SmpB (purple) and the tmRNA TLD (red). *Right*, Focus on the TLD nucleotides within 4 Å of SmpB.

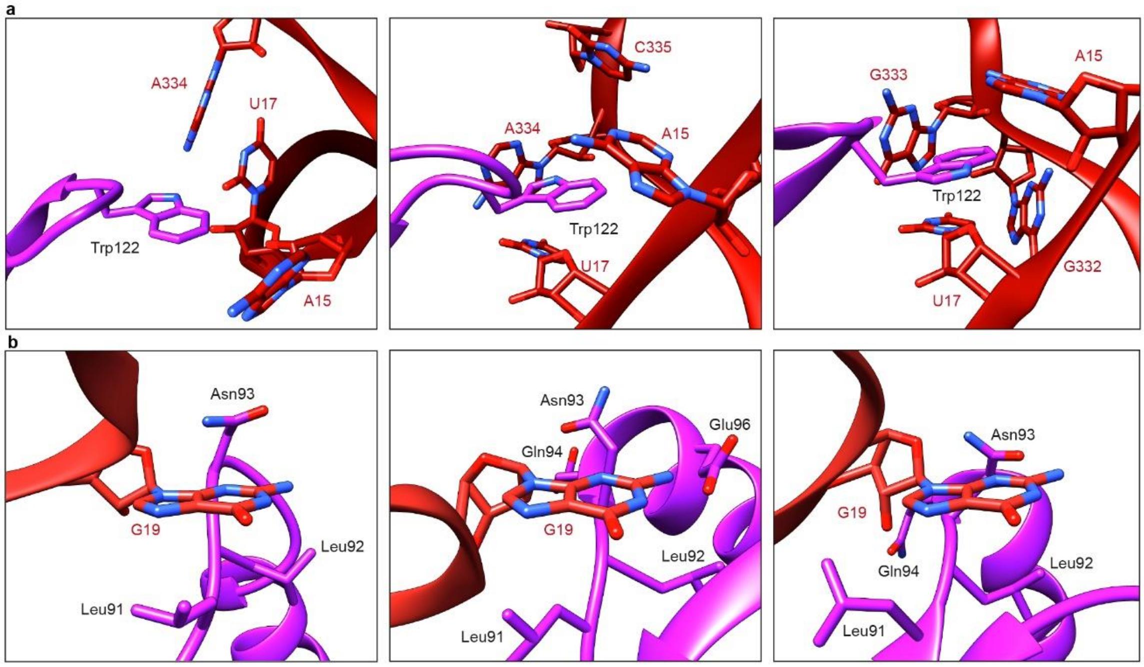

Supplementary Figure 9. Crucial interactions between tmRNA and SmpB are maintained throughout the *trans*-translation process. (a) SmpB's Trp122 is deeply embedded in a hydrophobic pocket delimited by tmRNA's U17 and A334. **(b)** The G19 nucleotide of tmRNA is tightly packed on the hydrophobic surface of SmpB. Shown are *left,* pre-accommodation; *middle,* accommodation; and *right,* translocation states. SmpB is purple, tmRNA is red, residues and nucleotides within 4 Å of each other are indicated

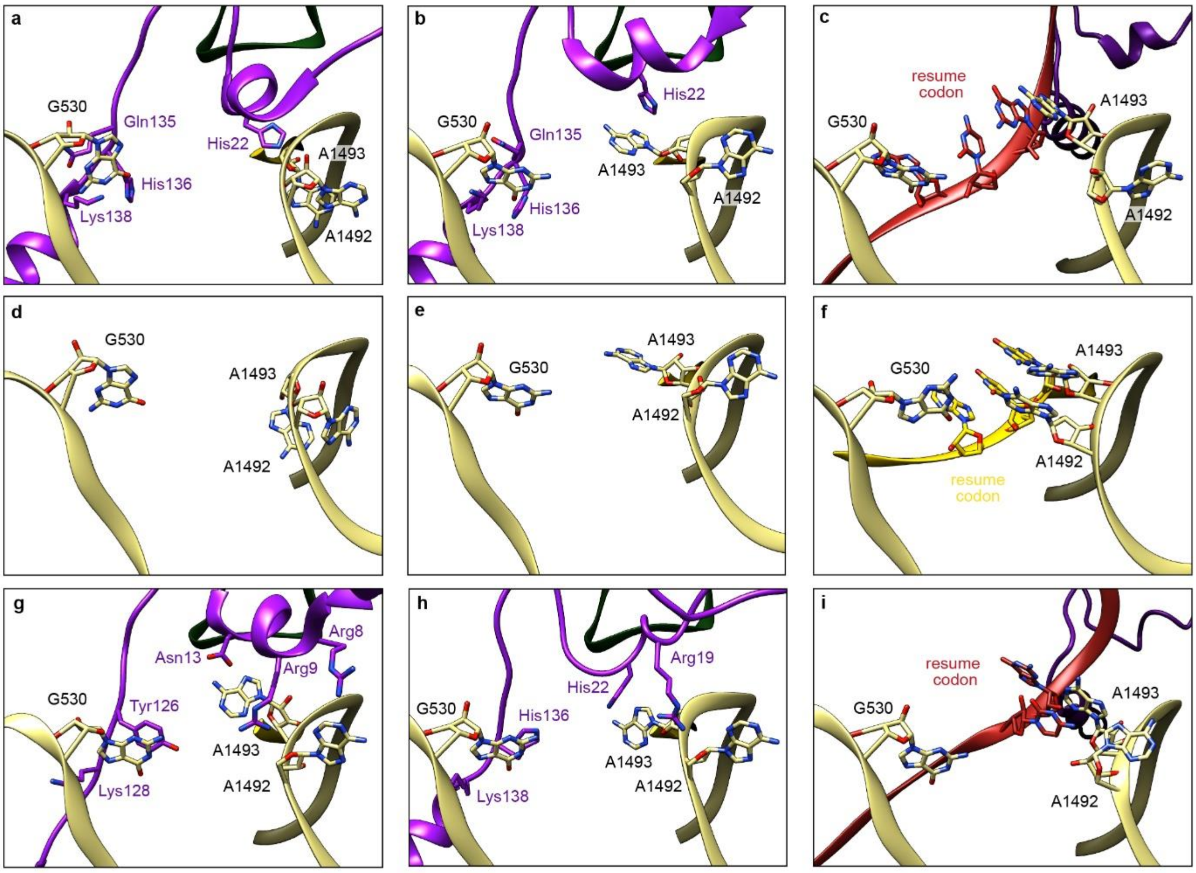

Supplementary Figure 10. Different conformation of the decoding centre. Close-up of the decoding centre (DC) in our **(a)** pre-accommodation, **(b)** accommodation, and **(c)** translocation states. **(d)** The DC of an empty *E. coli* ribosome[4] (PDB code 4YBB). **(e)** The DC of an *E. coli* ribosome with a tRNA in the P site, but an empty A site[5] (PDB code 5MDZ). **(f)** The DC in an *E. coli* ribosome with cognate tRNAs in the A and P sites[6] (PDB code 7K00). **(g)** The DC in a crystal structure of a complex containing a tmRNA fragment, SmpB, and EF-Tu bound to the *T. thermophilus* ribosome[2] (PDB code 4V8Q). **(h)** The DC in the accommodation state (PDB code 6Q97) described by Rae *et al.*[7] **(i)** Same as (h) but for the translocation state (PDB code 6Q98). In all panels, SmpB is purple, tmRNA is red, and the 16S rRNA is khaki. All residues and nucleotides that are within 4 Å of each other are indicated.

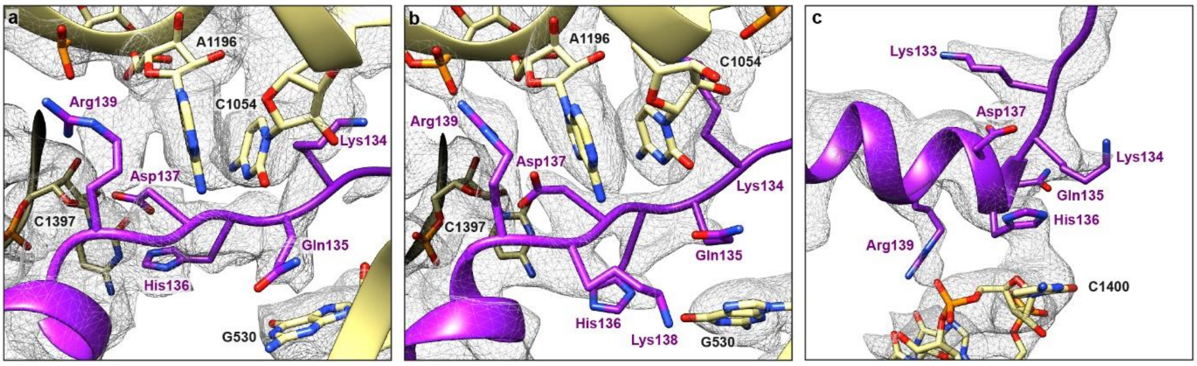

Supplementary Figure 11. Close-up of the region surrounding SmpB's His136. (a) In the pre-accommodation state, Lys134 and Arg139 both interact with the 16S rRNA and flank the stacked nucleotides C1054 and A1196. His136 and Asp137 form hydrogen bonds with C1397, and Gln135 is staked on G530. **(b)** These interactions are retained and even reinforced during the accommodation state, with Lys138 bonding with G530 and His136 interacting with both C1397 and G530. **(c)** His136 takes on a more crucial role during the translocation state. Its strong staking on C1440 as well as the hydrogen bonds formed between Arg139 and both C1399 and G1401 combine to place the SmpB C-terminal tail in the mRNA exit tunnel.

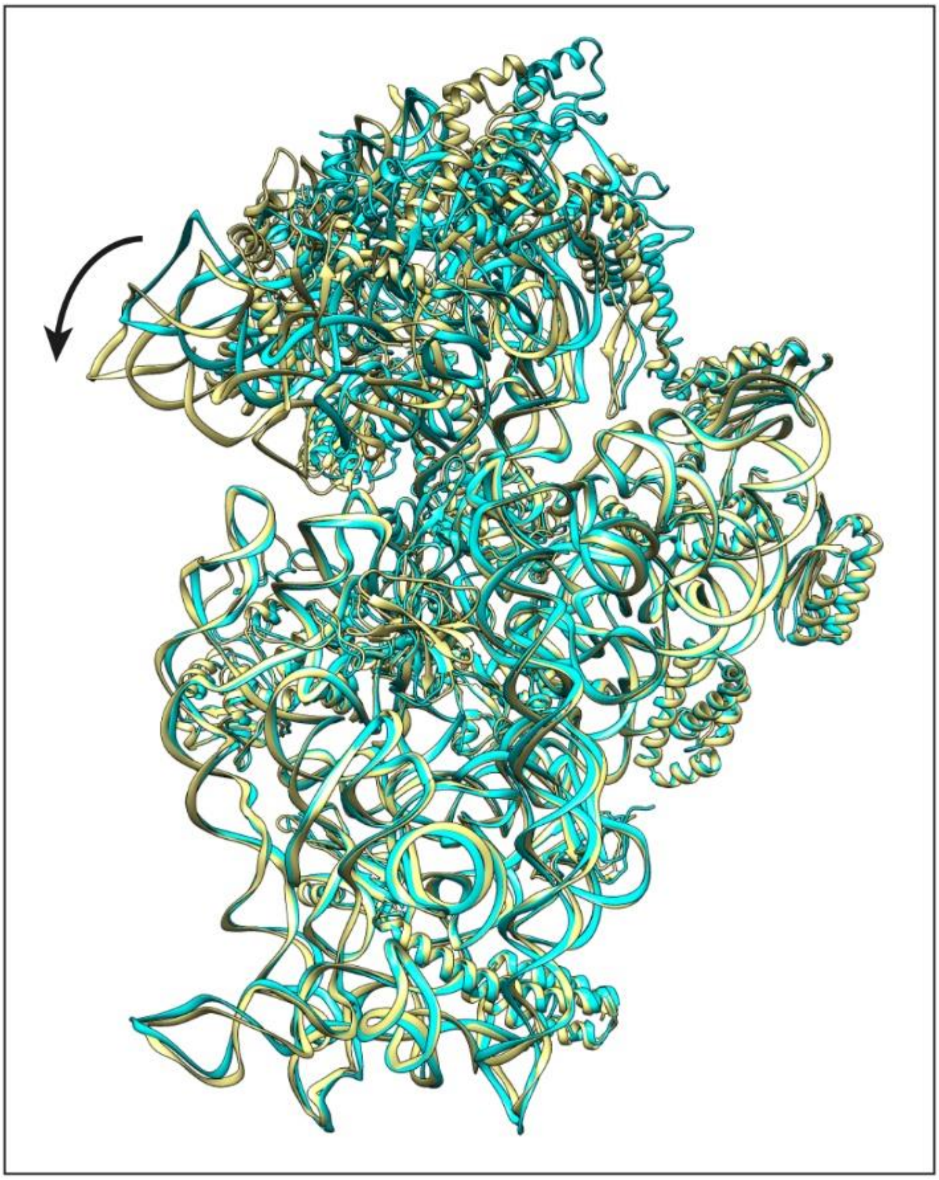

Supplementary Figure 12. Movement of the 30S subunit head during Ala-tmRNA·SmpB·EF-Tu·GDP pre-accommodation. Comparison between the 30S subunit in the pre-accommodation state (khaki) and that of an empty ribosome[8] (cyan; PDB code 4V4Q). The black arrow highlights the fact that when the quaternary complex made up of alanylated tmRNA, SmpB, EF-Tu, and GTP is bound, the small subunit head closes as if a cognate tRNA has been decoded.

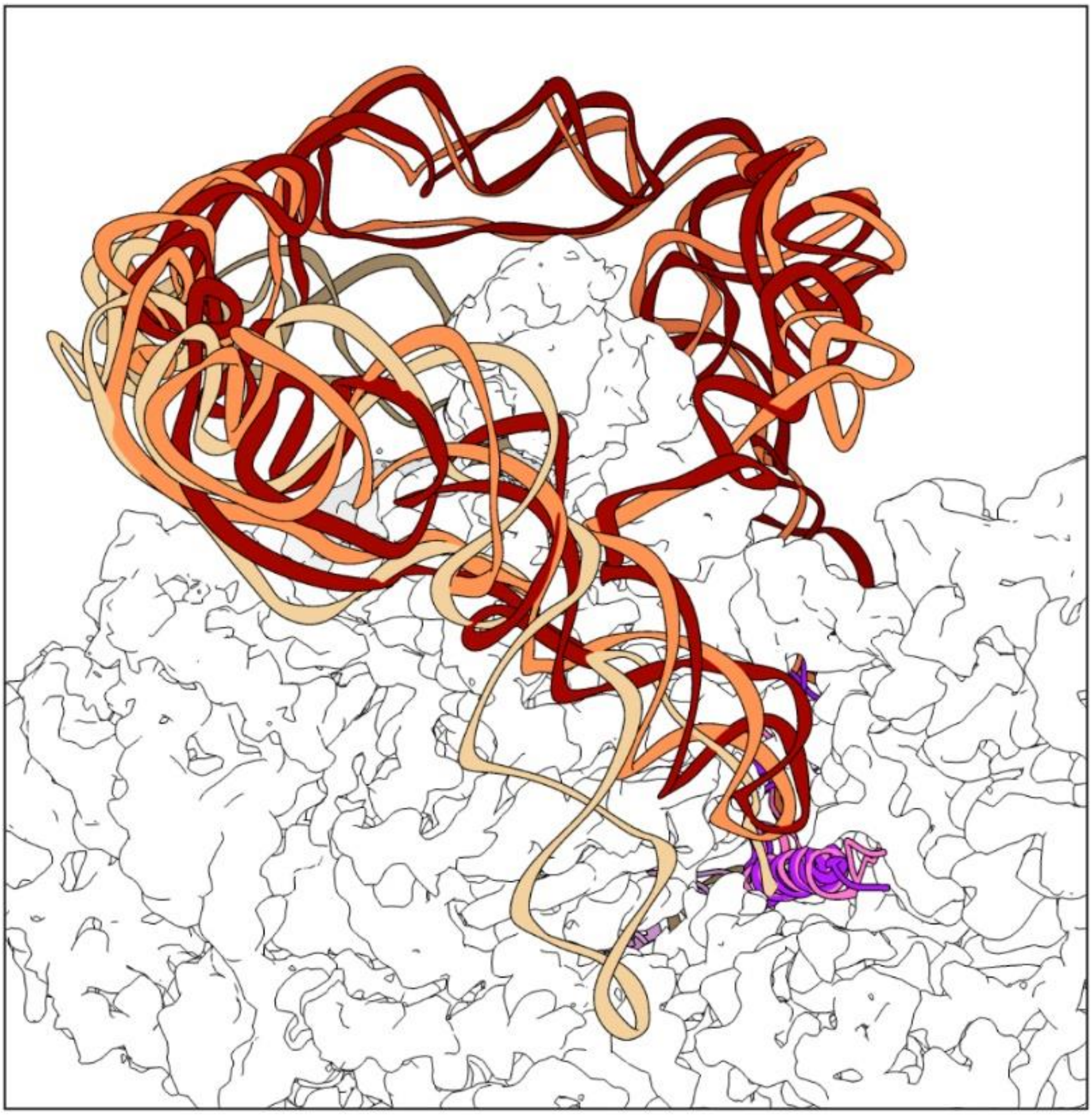

Supplementary Figure 13. Movement of the tmRNA pseudoknot ring and H5 stem-loop during *trans*-translation. Overlay of the atomic structures of the tmRNA-SmpB complex in the pre-accommodation state (tmRNA is red, SmpB is purple); the accommodation state (tmRNA is orange, SmpB is pink); and the translocation state (tmRNA is beige and SmpB is hidden behind the 30S head). The cryo-electron density map of 30S is shown as a white surface.

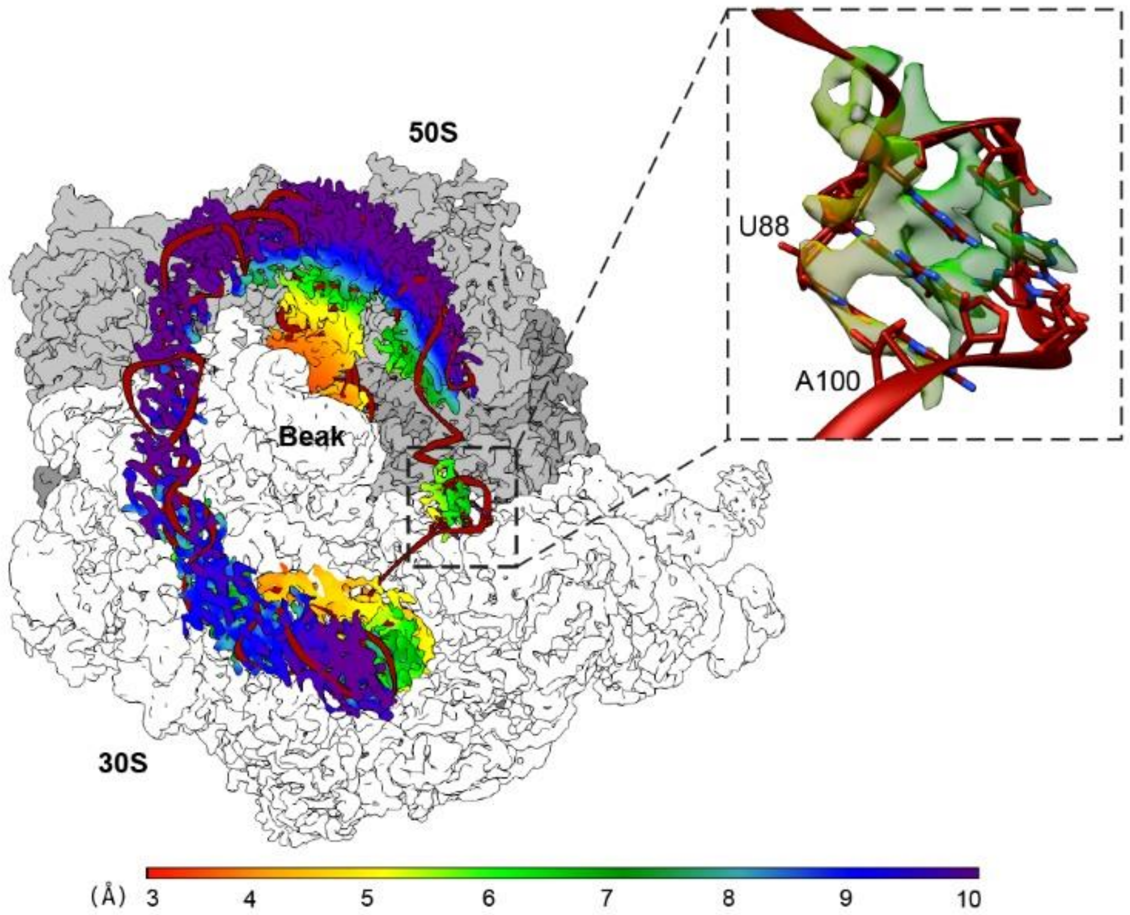

Supplementary Figure 14. In the accommodation state, the cryo-EM density confirms the presence of a hairpin in the tmRNA MLD. The zoom shows the dense region in the middle of the MLD, which is compatible with the presence of a hairpin between A88 and U100[9]. The 50S subunit is grey, the 30S is white, the tmRNA is red, and the cryo-electron density map around the MLD is shaded according to the local resolution as computed using ResMap[1].

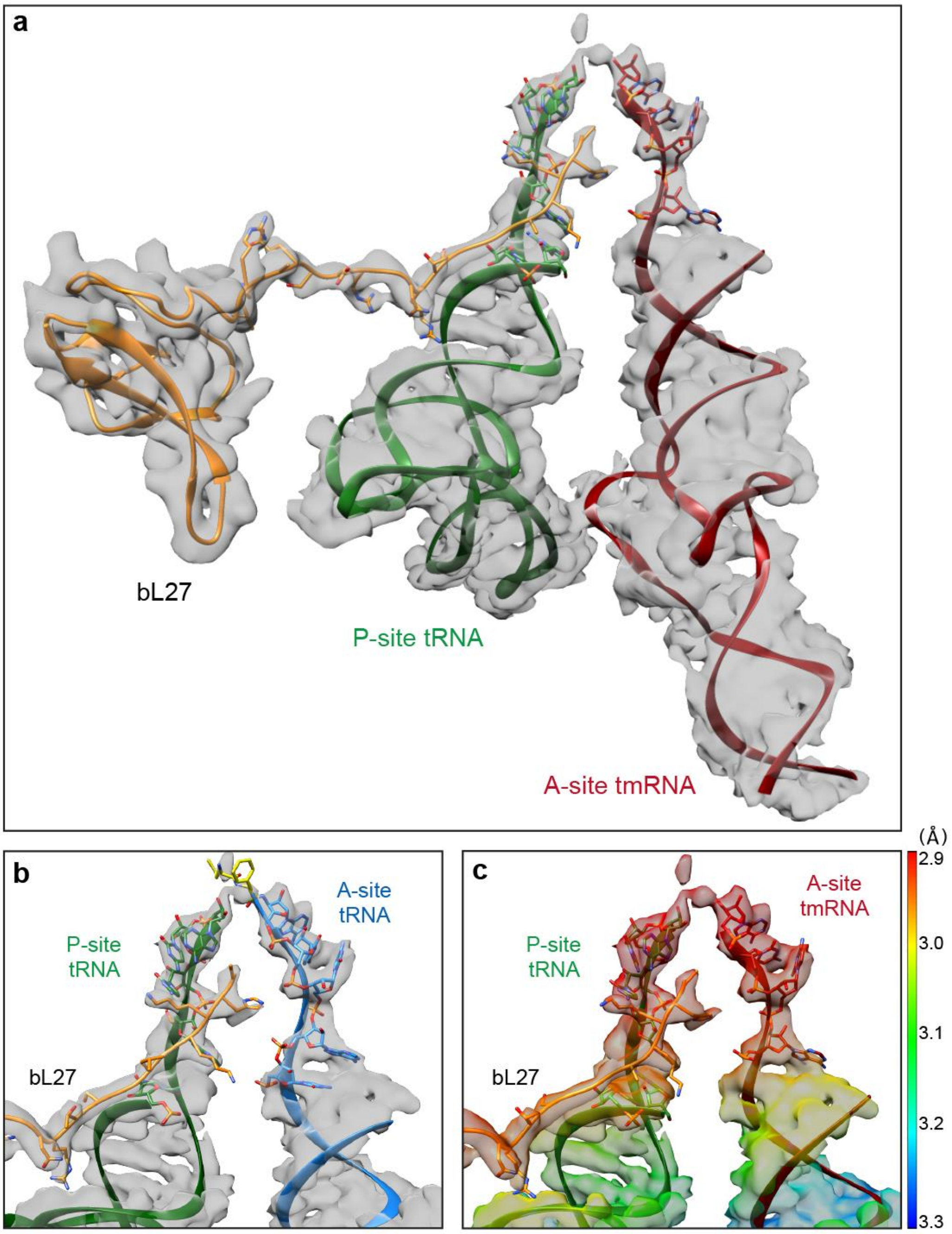

Supplementary Figure 15. Focus on the peptidyl transfer centre (PTC) during the accommodation state. (a) General view of the interaction between the ribosomal protein bL27 (orange), the P-site deacylated tRNA^{Phe} (green), and the dipeptidyl-tmRNA (red). The electron density map is shown in grey. **(b)** Close-up of the PTC in the post-catalysis state of canonical translation[10] (PDB code 1VY5) rigid-body fitted in our electron density map (grey). Here the dipeptidyl-tRNA is blue, the newly synthesized dipeptide is yellow, and the other colours are the same as in the other panels. **(c)** Close-up of the accommodation state. The electron density map is coloured according to the local resolutions as computed with ResMap[1] The TLD closely resembles an aminoacylated tRNA, and the flexible N-terminal arm of bL27 is in the same conformation as during canonical translation.

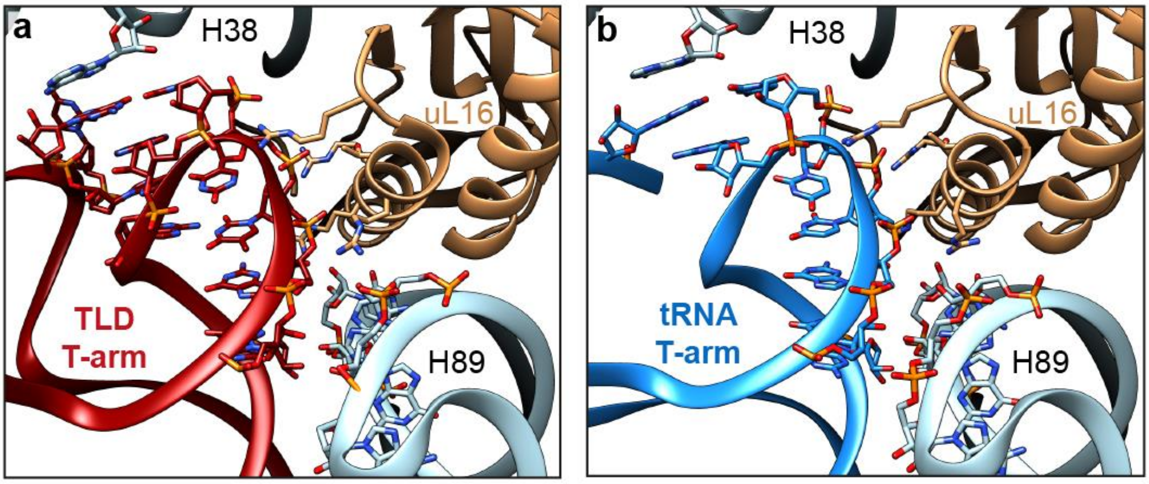

Supplementary Figure 16. Structural comparison between the T-arms of tmRNA and tRNA in the A site. (a) Close-up of the interaction between the tmRNA T-arm and the 50S ribosomal subunit. **(b)** Same, but for the tRNA T-arm[6] (PDB code 7K00). The tmRNA is red, tRNA is blue, uL16 is brown, and the 16S rRNA is light blue. To highlight the similarities between the two structures, the residues and nucleotides that are within 4 Å of each other are indicated.

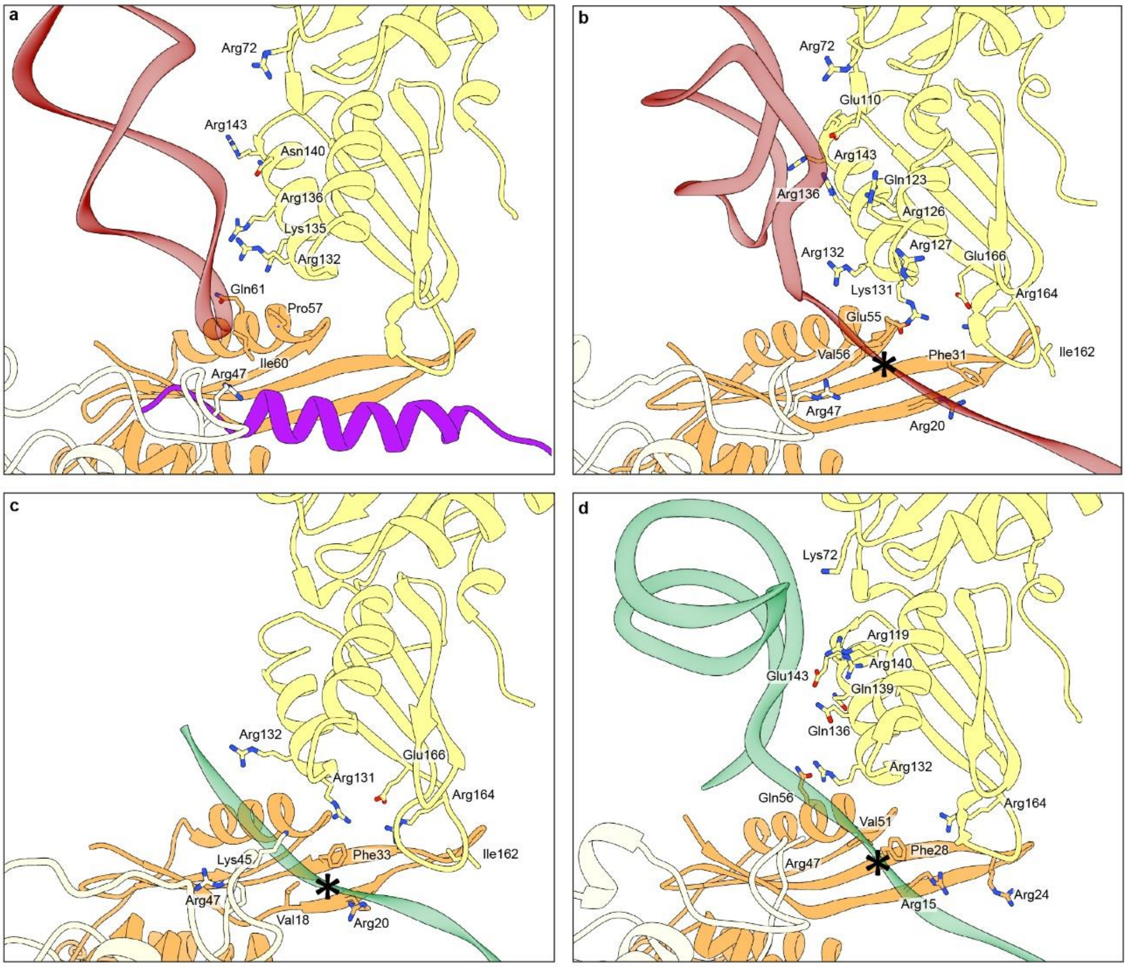

Supplementary Figure 17. Insight into the mechanism of action of the ribosomal helicase during *trans*-translation. (a) Close-up of the interactions between the H5 stem-loop and the ribosomal proteins uS3, uS4, and uS5 in the accommodation state. **(b)** Same, but for the translocation state. **(c)** Same, but for the single-stranded portion of a hairpin-containing mRNA[11] (PDB code 6BY1) **(d)** Same, but for the dnaX mRNA stem-loop bound to the *Thermus thermophilus* ribosome[12] (PDB code 5UQ7). SmpB is purple, tmRNA is red, mRNAs are green, and the ribosomal proteins uS3, uS4, and uS5 are khaki, orange, and white, respectively. The helicase residues within 4 Å of tmRNA or mRNAs are indicated. The stars represent the position of the proximal helicase active site, 11 nucleotides away from the P site[13]. To facilitate the comparison, all four structures are aligned on uS5.

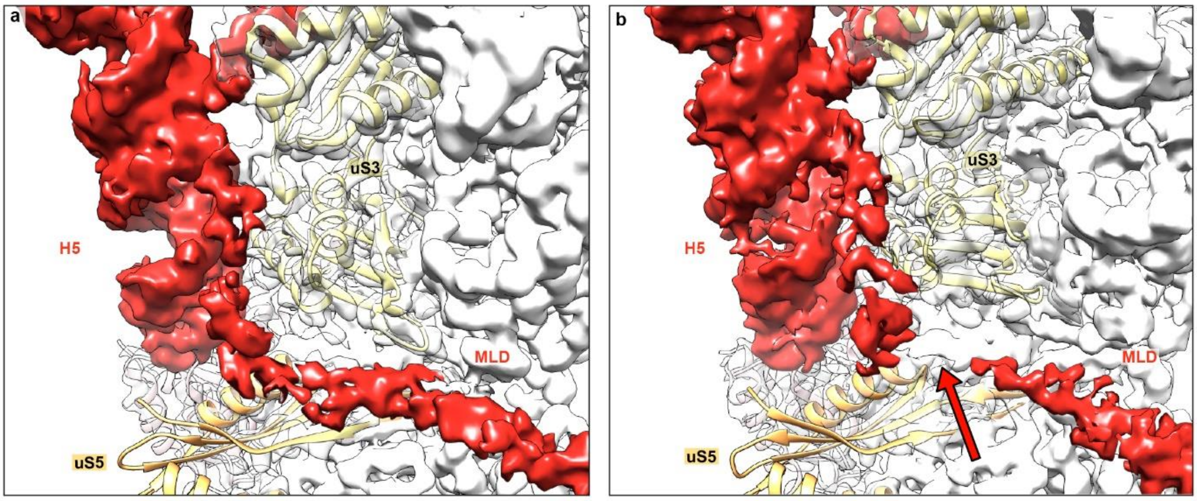

Supplementary Figure 18. Insight into the mechanism of action of the ribosomal helicase during the translocation steps. Close-up of the H5 stem-loop and MLD at the entrance of the mRNA channel in the **(a)** TRANS state and **(b)** TRANS* state. The electron density map of tmRNA is red and the 30S is white or transparent to allow for the localisation of the ribosomal protein uS3 and uS5 (khaki and orange, respectively). The arrow signals that in the TRANS* map, the MLD density is missing in the region of the proximal helicase active site[13].

Supplementary Tables

Supplementary Table 1. Cryo-EM data collection, refinement, and validation statistics.

	#1 PRE-ACC (EMD-11710) (PDB 7ABZ)	#2 ACC (EMD-11713) (PDB 7AC7)	#3 TRANS (EMDB-11717) (PDB 7ACJ)	#4 TRANS* (EMDB-11718) (PDB 7ACR)
**Data collection and processing**				
Magnification	105k	130k	130k	130k
Voltage (kV)	300	300	300	300
Electron exposure (e–/Å^2)	35	29	29	29
Defocus range (μm)	-1 to -3	-0.7 to -2	-0.7 to -2	-0.7 to -2
Pixel size (Å)	1.1 (fitted to 1.074)	1.04	1.04	1.04
Symmetry imposed	none	none	none	None
Micrographs collected (no.)	4,343	22,350	11,812	11,812
Micrographs used (no.)	3,143	21,917	11,433	11,433
Initial particle images (no.)	59,016	580,382	207,135	207,135
Final particle images (no.)	18,452	36,069	14,192	11,059
Map resolution (Å)				
FSC threshold: 0.143	3.21	3.08	3.20	3.44
FSC threshold: 0.5	4.00	3.49	4.16	5.00
Map resolution range (Å)	2.9-17.6	2.9-14.4	2.9-12.3	3.0-15.2
**Refinement**				
Initial models used (PDB code)	4YBB, 5AFI, 4V8Q	4YBB, 6Q97	4YBB, 6Q98	4YBB, 6Q98
Model resolution (Å)				
FSC threshold: 0.143	3.1	2.8	2.8	2.9
Map sharpening				
B factor (Å^2)	-42	-35	-24	-25
Model composition				
Non-hydrogen atoms	157,237	154,880	151,798	151,662
Protein residues	6,311	5,975	5,864	5,858
Nucleotide	5,017	5,017	4,920	4,920
Ligands	194	443	310	214
B factors mean (Å^2)				
Protein	111.73	98.05	117.32	147.69
Nucleotide	156.02	124.93	138.48	170.65
Ligand	93.89	72.87	84.93	92.48
RMS deviations				
Bond lengths (Å)	0.002	0.002	0.003	0.005
Bond angles (°)	0.648	0.494	0.567	0.594
Validation				
MolProbity score	2.15	1.71	1.99	2.01
Clashscore	16.22	10.27	13.20	14.14
Poor rotamers (%)	0.54	0.37	0.58	0.48
Ramachandran plot				
Favoured (%)	93.30	96.98	94.75	94.90
Allowed (%)	6.49	2.92	5.09	4.96
Disallowed (%)	0.21	0.10	0.16	0.14

Supplementary Table 2. 30S-head-to-body and 30S-body-to-50S rotations in ribosomal complexes.

Refs.	PDB ID	Body rotation (°)	Head rotation (°)	Head tilt (°)	tmRNA-SmpB location	tRNA location	Factors	Antibiotics	Organism	Resolution (Å)
Jenner et al. (2009)[14]	4V6F	0 / 1.3	0 / -0.5	0 / 0.7	-	A,P,E	-	-	Eco	3.1
PRE	7ABZ	0.3	0.1	1.5	pre A	P,E	EF-Tu•GDP	Kir	Eco	3.2#
ACC	7AC7	-1.3	1.5	0.7	A	P	-	-	Eco	3.1#
TRANS	7ACJ	-1.8	5.7	3.7	P	-	-	-	Eco	3.2#
TRANS*	7ACR	-1.2	13.9	12.2	P	-	-	-	Eco	3.4#
Carbone et al. (2020)[15]	7JT1	0.5	0.0	0.7	-	P	ArfB-1, ArfB-2	-	Eco	3.30#
Amiri and Noller (2019)[11]	6BY1	1.8 / 1.3	-0.1 / -0.3	1.0 / 1.0	-	A,P and A,P,E	Hairpin-containing mRNA	-	Eco	3.94
Fischer et al. (2015)[16]	5AFI	0.9	-0.8	0.3	-	pre A	EF-Tu•GDP	Kir	Eco	2.9
Watson et al. (2020)[6]	7K00	0.6	-0.2	0.6	-	A/P/E	-	Par	Eco	1.98#
Pulk et al. (2013)[17]	4V9O	2.8 / 3.8 / 4.0 / 6.7	6.1 / 7.1 / 5.3 / 11.4	0.8 / 0.2 / 2.1 / 3.3	-	-	EF-G•GDPCP	Vio	Eco	2.9
Noeske et al. (2015)[4]	4YBB	-0.6 /5.5	7.0 / 10.3	2.2 / 0.9	-	-	-	-	Eco	2.1
James et al. (2016)[5]	5MDZ	-1.8	1.5	1.6	-	P	-	-	Eco	3.1
Ramrath et al. (2013)[18]	4V7B	3.2	17.8	3.1	-	ap/P, pe/E	EF-G•GDP	Fus	Eco	6.8#
Ramrath et al. (2012)[19]	4V6T	6.0	17.0	11.5	P	E	EF-G	Fus	Eco	8.3#
Dunkle et al. (2011)[20]	4V9D	-0.1 / 8.9	0.2 / 3.5	0.6 / 0.9	-	P/E and P respectively	-	-	Eco	3.0
Li et al. (2015)[21]	3J9Z	-1.9	1.6	2.1	-	P,E	EF-G•GTP	-	Eco	3.6#
Li et al. (2015)[21]	3JA1	8.3	5.6	1.2	-	P/E	EF-G•GTP		Eco	3.6#
Rae et al. (2019)[7]	6Q97	-1.5	1.9	1.0	A	E**	-	-	Eco	3.9#
Rae et al. (2019)[7]	6Q98	-1.4	5.1	4.0	P	-	-	-	Eco	4.3#
Rae et al. (2019)[7]	6Q9A	-1.9	1.7	1.5	past E	P	-	-	Eco	3.7#
Rae et al. (2019)[7]	6Q95	-0.9	2.3	1.0	A	P,E	-	-	Tth	3.7#
Neubauer et al. (2012)[2]	4V8Q	-0.1	0.7	0.5	A*	P,E	EF-Tu•GDP	Kir	Tth	3.1
Zhou et al. (2014)[22]	4W29	3.0 / 3.0	20.1 / 20.4	3.2 / 2.8	-	AP/AP,pe/E	EF-G•GDP	Fus	Tth	3.80
Schuwirth et al. (2005)[8]	4V4Q	-1.5 / -2.6	15.4 / 6.7	1.1 / 2.6	-	-	-	-	Tth	3.46
Polikanov et al. (2014)[10]	1VY5	-0.9 / 1.0	0.9 / -0.5	0.6 / 0.9	-	A,P,E	-	-	Tth	2.55
Voorhees et al. (2010)[3]	4V5L	0.6	-1.0	0.4	-	A,P,E	EF-Tu•GDPCP		Tth	3.10
Hong et al. (2018)[23]	5VPP	-0.8 / 0.3	19.0 / 19.2	3.3 / 3.0		e*/E			Tth	3.9

Calculations are based on Nguyen and Whitford[24], and we used the same structure (PDB 4V6F) as the reference configuration[14] (see first line). For the movements, when multiple ribosomes are present in the asymmetric unit, all values are shown. Key: Fus, fusidic acid; Kir, kirromycin; Par, paromomycin; Vio, viomycin; Eco, *Escherichia coli*; Tth, *Thermus thermophilus*; *, only an engineered TLD is present; **, P-site fMet-NH-tRNA is present in the atomic model but not in the cryo-EM map; and #, structure derived from cryo-EM.

Supplementary Table 3. Bacterial strains, plasmids, and synthetic sequences.

Strain name	Description	Source
E. coli MG1655	Strain used for ribosome purification	CGSC (The Coli Genetic Stock Center)
E. coli BL21(DE3)*ΔssrA*	Strain used to purify SmpB protein	[25]

Plasmid name	Description	Source
pUC19-ala-tRNA	Plasmid to produce Alanine specific tRNA	[26]
pQE30-AlaRS	Plasmid to express and purify alanyl-tRNA synthetase	[27]
pQE30-PheRS	Plasmid to express and purify phenylalanyl-tRNA synthetase	[27]
pQE60-EF-G	Plasmid to express and purify EF-G	[27]
pABA-RNR_D280N	Plasmid to express and purify the mutated RNase R (D280N)	[28]

DNA name	Description	Sequence	Source
non-stop mRNA	Small non-stop mRNA to stall ribosomes	5′-<u>AGGAGG</u>UGAGGUUUU-3′	Thermo Fisher Scientific
tRNA-phe	Phenylalanine-specific tRNA isolated from E. coli MRE 600		Sigma
Forward_SmpB-N*de*I	Primer#1	5'-TCACGACGCATATGACGAAG	[29]
Reverse_SmpB-X*ho*I	Primer#2	5'-ACTCGAGACGGTGGGCGTTTTTC	[29]

Supplementary References

1. Kucukelbir, A., Sigworth, F. J. & Tagare, H. D. Quantifying the local resolution of cryo-EM density maps. *Nat. Methods* **11**, 63–65 (2014).
2. Neubauer, C., Gillet, R., Kelley, A. C. & Ramakrishnan, V. Decoding in the absence of a codon by tmRNA and SmpB in the ribosome. *Science* **335**, 1366–1369 (2012).
3. Voorhees, R. M., Schmeing, T. M., Kelley, A. C. & Ramakrishnan, V. The Mechanism for Activation of GTP Hydrolysis on the Ribosome. *Science* **330**, 835–838 (2010).
4. Noeske, J. et al. High-resolution structure of the Escherichia coli ribosome. *Nat. Struct. Mol. Biol.* **22**, 336–341 (2015).
5. James, N. R., Brown, A., Gordiyenko, Y. & Ramakrishnan, V. Translational termination without a stop codon. *Science* **354**, 1437–1440 (2016).
6. Watson, Z. L. et al. Structure of the bacterial ribosome at 2 Å resolution. *Elife* **9**, (2020).
7. Rae, C. D., Gordiyenko, Y. & Ramakrishnan, V. How a circularized tmRNA moves through the ribosome. *Science* **363**, 740–744 (2019).
8. Schuwirth, B. S. et al. Structures of the bacterial ribosome at 3.5 A resolution. *Science* **310**, 827–834 (2005).
9. Felden, B. et al. Probing the structure of the Escherichia coli 10Sa RNA (tmRNA). *RNA* **3**, 89–103 (1997).
10. Polikanov, Y. S., Steitz, T. A. & Innis, C. A. A proton wire to couple aminoacyl-tRNA accommodation and peptide-bond formation on the ribosome. *Nat. Struct. Mol. Biol.* **21**, 787–793 (2014).
11. Amiri, H. & Noller, H. F. Structural evidence for product stabilization by the ribosomal mRNA helicase. *RNA* **25**, 364–375 (2019).
12. Zhang, Y., Hong, S., Ruangprasert, A., Skiniotis, G. & Dunham, C. M. Alternative Mode of E-Site tRNA Binding in the Presence of a Downstream mRNA Stem Loop at the Entrance Channel. *Structure* **26**, 437-445.e3 (2018).
13. Amiri, H. & Noller, H. F. A tandem active site model for the ribosomal helicase. *FEBS Lett.* **593**, 1009–1019 (2019).

14. Jenner, L. B., Demeshkina, N., Yusupova, G. & Yusupov, M. Structural aspects of messenger RNA reading frame maintenance by the ribosome. *Nat. Struct. Mol. Biol.* **17**, 555–560 (2010).

15. Carbone, C. E., Demo, G., Madireddy, R., Svidritskiy, E. & Korostelev, A. A. ArfB can displace mRNA to rescue stalled ribosomes. *Nat. Comm.* **11**, 5552 (2020).

16. Fischer, N. et al. Structure of the E. coli ribosome-EF-Tu complex at <3 Å resolution by Cs-corrected cryo-EM. *Nature* **520**, 567–570 (2015).

17. Pulk, A. & Cate, J. H. D. Control of ribosomal subunit rotation by elongation factor G. *Science* **340**, 1235970 (2013).

18. Ramrath, D. J. F. et al. Visualization of two transfer RNAs trapped in transit during elongation factor G-mediated translocation. *Proc Natl. Acad. Sci. U.S.A.* **110**, 20964–20969 (2013).

19. Ramrath, D. J. F. et al. The complex of tmRNA-SmpB and EF-G on translocating ribosomes. *Nature* **485**, 526–529 (2012).

20. Dunkle, J. A. et al. Structures of the bacterial ribosome in classical and hybrid states of tRNA binding. *Science* **332**, 981–984 (2011).

21. Li, W. et al. Activation of GTP hydrolysis in mRNA-tRNA translocation by elongation factor G. *Sci. Adv.* **1**, (2015).

22. Zhou, J., Lancaster, L., Donohue, J. P. & Noller, H. F. How the ribosome hands the A-site tRNA to the P site during EF-G-catalyzed translocation. *Science* **345**, 1188–1191 (2014).

23. Hong, S. et al. Mechanism of tRNA-mediated +1 ribosomal frameshifting. *Proc. Natl. Acad. Sci. U.S.A.* **115**, 11226–11231 (2018).

24. Nguyen, K. & Whitford, P. C. Steric interactions lead to collective tilting motion in the ribosome during mRNA–tRNA translocation. *Nat. Comm.* **7**, 10586 (2016).

25. Cougot, N. et al. Visualizing compaction of polysomes in bacteria*. J. Mol. Biol.* **426**, 377–388 (2014).

26. Gillet, R. & Felden, B. Transfer RNA(Ala) recognizes transfer-messenger RNA with specificity; a functional complex prior to entering the ribosome? *EMBO J.* **20**, 2966–2976 (2001).

27. Shimizu, Y. et al. Cell-free translation reconstituted with purified components. *Nat. Biotechnol.* **19**, 751–755 (2001).

28. Matos, R. G., Barbas, A. & Arraiano, C. M. RNase R mutants elucidate the catalysis of structured RNA: RNA-binding domains select the RNAs targeted for degradation. *Biochem. J.* **423**, 291–301 (2009).

29. Guyomar, C. et al. Reassembling green fluorescent protein for in vitro evaluation of trans-translation. *Nucleic Acids Res.* **48**, e22 (2020).
