# Supplementary material for: Structures of tmRNA and SmpB as they transit through the ribosome
Source: Nat Commun. 2021 Aug 13;12:4909. doi: 10.1038/s41467-021-24881-4 (PMC8363625; doi:10.1038/s41467-021-24881-4)
Supplement: Supplementary file 4 — Description of additional supplementary files [file 41467_2021_24881_MOESM4_ESM.docx]

Description of additional supplementary files

Title: Supplementary Movie 1.

Description: Focus on the opening of bridges B1b and B1c during the transition between the TRANS and TRANS* states
