# Supplementary material for: Structures of tmRNA and SmpB as they transit through the ribosome
Source: Nat Commun. 2021 Aug 13;12:4909. doi: 10.1038/s41467-021-24881-4 (PMC8363625; doi:10.1038/s41467-021-24881-4)
Supplement: Supplementary file 5 — Reporting summary [file 41467_2021_24881_MOESM5_ESM.pdf]

nature portfolio

Corresponding author(s): Reynald GILLET, Emmanuel GIUDICE

Last updated by author(s): 2021/06/21

Reporting Summary

Nature Portfolio wishes to improve the reproducibility of the work that we publish. This form provides structure for consistency and transparency in reporting. For further information on Nature Portfolio policies, see our Editorial Policies and the Editorial Policy Checklist.

Statistics

For all statistical analyses, confirm that the following items are present in the figure legend, table legend, main text, or Methods section.

n/a	Confirmed	
☒	☐	The exact sample size (*n*) for each experimental group/condition, given as a discrete number and unit of measurement
☒	☐	A statement on whether measurements were taken from distinct samples or whether the same sample was measured repeatedly
☒	☐	The statistical test(s) used AND whether they are one- or two-sided<br>*Only common tests should be described solely by name; describe more complex techniques in the Methods section.*
☒	☐	A description of all covariates tested
☒	☐	A description of any assumptions or corrections, such as tests of normality and adjustment for multiple comparisons
☒	☐	A full description of the statistical parameters including central tendency (e.g. means) or other basic estimates (e.g. regression coefficient) AND variation (e.g. standard deviation) or associated estimates of uncertainty (e.g. confidence intervals)
☒	☐	For null hypothesis testing, the test statistic (e.g. *F*, *t*, *r*) with confidence intervals, effect sizes, degrees of freedom and *P* value noted<br>*Give P values as exact values whenever suitable.*
☒	☐	For Bayesian analysis, information on the choice of priors and Markov chain Monte Carlo settings
☒	☐	For hierarchical and complex designs, identification of the appropriate level for tests and full reporting of outcomes
☒	☐	Estimates of effect sizes (e.g. Cohen's *d*, Pearson's *r*), indicating how they were calculated

Our web collection on statistics for biologists contains articles on many of the points above.

Software and code

Policy information about availability of computer code

Data collection	Cryo-EM images were recorded using SerialEM v3.6.14
Data analysis	Movies were corrected for the effects of drift and beam-induces motion using MotionCor2 v1.0.6. Contrast transfer function parameters were estimated using Gctf v1.18. Particles were semi-automatically selected and subjected to two rounds of 2D classification using Cryosparc v2.12 in order to discard defective particles. All subsequent data processing was performed in Relion 3.1 Beta. Local resolutions were estimated using Resmap v1.1.4. Atomic models were build using Coot v0.9 and Phenix v1.18.2, with the exception of the MLD and pseudoknots which were first adjusted with MDFF using VMD 1.8.2, NAMD v1.13 and the CHARMM36 force field. Models were evaluated with MolProbity v4.5.1 and the remaining analysis and the illustrations were done using UCSF-Chimera v1.13.1.

For manuscripts utilizing custom algorithms or software that are central to the research but not yet described in published literature, software must be made available to editors and reviewers. We strongly encourage code deposition in a community repository (e.g. GitHub). See the Nature Portfolio guidelines for submitting code & software for further information.

Data

Policy information about availability of data

All manuscripts must include a data availability statement. This statement should provide the following information, where applicable:

- Accession codes, unique identifiers, or web links for publicly available datasets
- A description of any restrictions on data availability
- For clinical datasets or third party data, please ensure that the statement adheres to our policy

All of the data supporting the finding of this study are available within the present paper and in the supplementary materials. The atomic coordinates and electron density maps have been deposited in the EMDB and PDB under the following accession codes, respectively: EMD-11710 [https://www.ebi.ac.uk/pdbe/entry/emdb/

EMD-11710] and 7ABZ [http://doi.org/10.2210/pdb7abz/pdb] (pre-accommodated state); EMD-11713 [https://www.ebi.ac.uk/pdbe/entry/emdb/EMD-11713] and 7AC7 [http://doi.org/10.2210/pdb7ac7/pdb] (accommodated state); EMD-11717 [https://www.ebi.ac.uk/pdbe/entry/emdb/EMD-11717] and 7ACJ [http://doi.org/10.2210/pdb7acj/pdb] (translocated state); and EMD-11718 [https://www.ebi.ac.uk/pdbe/entry/emdb/EMD-11718] and 7ACR [http://doi.org/10.2210/pdb7acr/pdb] (post-translocated intermediate state).
Additionally, the following atomic coordinates were used as initial coordinates for the model building and/or for comparison: (1VY5 [http://doi.org/10.2210/pdb1vy5/pdb], 3JA1 [http://doi.org/10.2210/pdb3ja1/pdb], 3J9Z [http://doi.org/10.2210/pdb3j9z/pdb], 4V4Q [http://doi.org/10.2210/pdb4v4q/pdb], 4V5L [http://doi.org/10.2210/pdb4v5l/pdb], 4V6F [http://doi.org/10.2210/pdb4v6f/pdb], 4V6T [http://doi.org/10.2210/pdb4v6t/pdb], 4V7B [http://doi.org/10.2210/pdb4v7b/pdb], 4V8Q [http://doi.org/10.2210/pdb4v8q/pdb], 4V9D [http://doi.org/10.2210/pdb4v9d/pdb], 4V9O [http://doi.org/10.2210/pdb4v9o/pdb], 4W29 [http://doi.org/10.2210/pdb4w29/pdb], 4YBB [http://doi.org/10.2210/pdb4ybb/pdb], 5AFI [http://doi.org/10.2210/pdb5AFI/pdb], 5MDZ [http://doi.org/10.2210/pdb5mdz/pdb], 5UQ7 [http://doi.org/10.2210/pdb5uq7/pdb], 5VPP [http://doi.org/10.2210/pdb5vpp/pdb], 6BY1 [http://doi.org/10.2210/pdb6BY1/pdb], 6Q9A [http://doi.org/10.2210/pdb6q9a/pdb], 6Q95 [http://doi.org/10.2210/pdb6q95/pdb], 6Q97 [http://doi.org/10.2210/pdb6q97/pdb], 6Q98 [http://doi.org/10.2210/pdb6q98/pdb], 6ZTJ [http://doi.org/10.2210/pdb6ztj/pdb], 7JT1 [http://doi.org/10.2210/pdb7JT1/pdb], 7K00 [http://doi.org/10.2210/pdb7K00/pdb])

Field-specific reporting

Please select the one below that is the best fit for your research. If you are not sure, read the appropriate sections before making your selection.

☒ Life sciences ☐ Behavioural & social sciences ☐ Ecological, evolutionary & environmental sciences

For a reference copy of the document with all sections, see nature.com/documents/nr-reporting-summary-flat.pdf

Life sciences study design

All studies must disclose on these points even when the disclosure is negative.

Sample size	Sample size was determined for obtaining enough number of particles to reconstruct high quality 3D maps. Three independent datasets containing 3,143, 10,484, and 11,433 movies were recorded. This resulted in 59,016 ((dataset 1), 373, 247 (dataset 2), and 207, 135 particles (dataset 3) which were sufficient to obtain high resolution structure as per standard single particle analyses procedures.
Data exclusions	Electron micrographs showing signs of drift or astigmatism were discard. Particles in poorly resolved 2D/3D classes were excluded as per standard single particle analyses procedure.
Replication	Multiple rounds of structural refinement were performed on the different dataset, and they converged to the same density maps.
Randomization	The single particle analysis was performed as per standard procedure, using Relion and the "gold standard" FSC procedure, meaning that during the map refinement the data set is divide into two random halves, and two sets of model parameters are refined separately. Further than that the study does not involve to randomly allocate samples into experimental groups because it focus on a specific macromolecular complex.
Blinding	Blinding is not relevant for this study because we are focusing on a specific macromolecular complex.

Reporting for specific materials, systems and methods

We require information from authors about some types of materials, experimental systems and methods used in many studies. Here, indicate whether each material, system or method listed is relevant to your study. If you are not sure if a list item applies to your research, read the appropriate section before selecting a response.

Materials & experimental systems

n/a	Involved in the study
☒	☐ Antibodies
☒	☐ Eukaryotic cell lines
☒	☐ Palaeontology and archaeology
☒	☐ Animals and other organisms
☒	☐ Human research participants
☒	☐ Clinical data
☒	☐ Dual use research of concern

Methods

n/a	Involved in the study
☒	☐ ChIP-seq
☒	☐ Flow cytometry
☒	☐ MRI-based neuroimaging
